# ACTsmart – development and feasibility of digital Acceptance and Commitment Therapy for adults with chronic pain

**DOI:** 10.1038/s41746-020-0228-4

**Published:** 2020-02-13

**Authors:** Charlotte Gentili, Vendela Zetterqvist, Jenny Rickardsson, Linda Holmström, Laura E. Simons, Rikard K. Wicksell

**Affiliations:** 1 Functional Unit Behavioral Medicine, Function Area Medical Psychology, Karolinska University Hospital, Stockholm, Sweden; 2 Department of Clinical Neuroscience, Karolinska Institutet, Stockholm, Sweden; 3 Department of Neuroscience, Uppsala University, Uppsala, Sweden; 4 Department of Women’s and Children’s Health Karolinska Institutet, Stockholm, Sweden; 5 Department of Anesthesiology, Perioperative and Pain Medicine, Stanford University School of Medicine, Palo Alto, CA USA

**Keywords:** Lifestyle modification, Quality of life

## Abstract

Accessibility of evidence-based behavioral health interventions is one of the main challenges in health care and effective treatment approaches are not always available for patients that would benefit from them. Digitization has dramatically changed the health care landscape. Although mHealth has shown promise in addressing issues of accessibility and reach, there is vast room for improvements. The integration of technical innovations and theory driven development is a key concern. Digital solutions developed by industry alone often lack a clear theoretical framework and the solutions are not properly evaluated to meet the standards of scientifically proven efficacy. On the other hand, mHealth interventions developed in academia may be theory driven but lack user friendliness and are commonly technically outdated by the time they are implemented in regular care, if they ever are. In an ongoing project aimed at scientific innovation, the mHealth Agile Development and Evaluation Lifecycle was used to combine strengths from both industry and academia in the development of ACTsmart – a smartphone-based Acceptance and Commitment Therapy treatment for adult chronic pain patients. The present study describes the early development of ACTsmart, in the process of moving the product from alpha testing to a clinical trial ready solution.

## Introduction

For many health conditions, such as chronic pain, access to evidence-based behavioral health interventions is limited due to geographical and financial reasons. Treatments that may be beneficial to restore or improve functioning are not always available. Digital solutions may contribute to more successful health care by increasing access and reach, with effects comparable to those in face-to-face treatment.^1^ Also, digital solutions provide the possibility of collecting both passive (system generated) and self-reported continuous data unbiased by retrospective recall, which may enable the aggregation of key information for further development of both treatment models and technical solutions.

Most digital health interventions developed within industry lack theory-based strategies known to drive behavior change,^2–4^ evidence-based content,^5–8^ and systematic efficacy-testing.^9^ In contrast, interventions developed within academia are usually derived from behavioral theory and evaluated scientifically.^10^ Unfortunately, methods such as randomized controlled trials are time-consuming and costly, which implies that the academic approach is less flexible than commercial mHealth development processes that utilize repeated rapid cycles of fine-tuning based on user feedback.^11^ The lengthy process of efficacy testing also prevents rapid dissemination, and novel digital interventions developed within academia are therefore at risk of being technically outdated when implemented in routine health care. Moreover, poor retention rates are common in digital solutions^12^ and negatively influence effect sizes.^13^

Chronic pain affects 18–30% of the adult population.^14^ Many individuals display significant reductions in daily functioning and quality of life and chronic pain is associated with elevated risks of insomnia, depression, suicidality, and anxiety.^14,15^ Cognitive, behavioral, and emotional factors play an important role in the maintenance of pain-related impairment^16^ and there is broad consensus concerning the utility of behavioral treatments for chronic pain.^17,18^ A vast number of smartphone applications targeting persons suffering with chronic pain have been developed during the past decade. However – as concluded in a review presenting 111 different digital pain applications^19^ – “Pain apps appear to be able to promise pain relief without any concern for the effectiveness of the product, or for possible adverse effects of product use”. In a more recent systematic review^20^ the problem raised in the 2011 review remains with the majority of the apps being simplistic; they lack involvement of health care professionals in the development, have no rigorous testing for efficacy on pain-related health outcomes, and lack a theoretical or evidence-based framework.^20^ Acceptance and Commitment Therapy (ACT; as first described by Hayes, et al.^21^) has strong empirical support for chronic pain.^22,23^ Despite the scientific support, access to ACT treatment is still limited, in part because treatment is usually provided face-to-face by therapists with specific training.

mHealth interventions can improve access and reach of effective behavioral-based pain treatment to those suffering with chronic pain. The digital format makes it possible to use automated and tailored messages, to send reminders and provide instant feedback as well as to collect both passive and self-reported data unbiased by retrospective recall, which allows for close follow up and research on treatment.^24^ To ascertain that the intervention is user friendly, effective, and up to date, there is a need for an approach that combines the strengths of the industry’s rapid development process and academia’s theory-based approach and efficacy testing. The mHealth Agile Development & Evaluation Lifecycle^25^ provides a framework for rapid and sustainable mHealth development, evaluation, and implementation.

The overall objective of the present project was to develop a digital solution (ACTsmart) that is user friendly, flexible, effective, accepted by the users, solicits retention, and is designed for continuous data collection while following individual participants in their daily life. The development team applied the mHealth Agile Development & Evaluation Lifecycle to promote continuous development, fine-tuning of treatment content, and data driven decision-making. Development is based on a series of short iterations, with alpha testing on a small sample of end-users followed by multiple re-iterations and testing until a satisfactory level was reached, which was subsequently beta tested on a sample of naïve end-users.

The present study describes phase one and two (alpha and beta testing) of the lifecycle. The aim of the study was twofold. Firstly, to document the development process (alpha and beta testing) and the gained insights, to make this knowledge available to other developers and scientists. Secondly, to scientifically evaluate and optimize ACTsmart to make it a clinical trial ready solution. In the Beta-testing the following feasibility aspects were evaluated (1) if ACTsmart was accepted by users (patients and therapists) as a means to deliver treatment, (2) to what extent patients interacted with the solution, and (3) if an ACTsmart delivered treatment was practically and technically feasible for patients and therapists.

## Results

For description of features, functionality and screenshots of the ACTsmart patient interface, please see Methods.

### Alpha testing (phase 1)

**Fig. 1:**
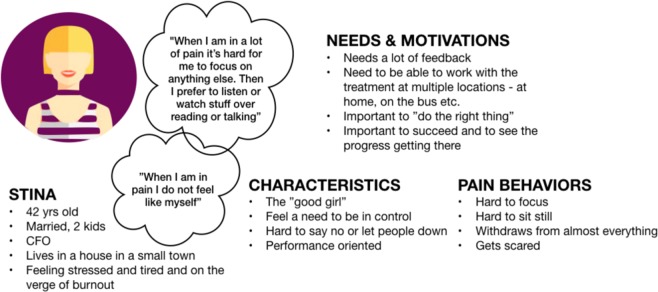
Persona generated after multiple initial end-user interviews (translated from Swedish to English).

In total, 15 individuals – nine chronic pain patients and six therapists – participated in the alpha testing of development phase 1. The alpha tests focused on *user friendliness* and *comprehensibility*. Figure 1 shows one of three patient personas that were generated from early user experience (UX) interviews describing demographic information, needs and motivations, characteristics and pain behaviors. Personas help designers create an understanding of the potential end users, and keep the end users in mind during the design process.

**Fig. 2:**
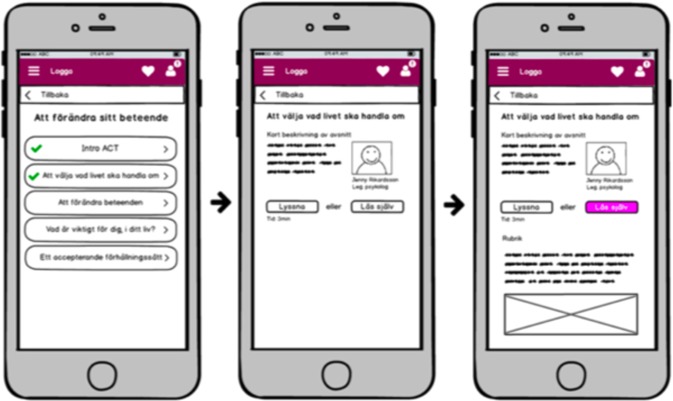
Paper sketches of patient interface used in alpha testing (the native version).

Figure 2 shows paper sketches that were used in alpha testing of the patient interface. Each alpha test was documented and used to guide following iterations of the tested function.

**Fig. 3:**
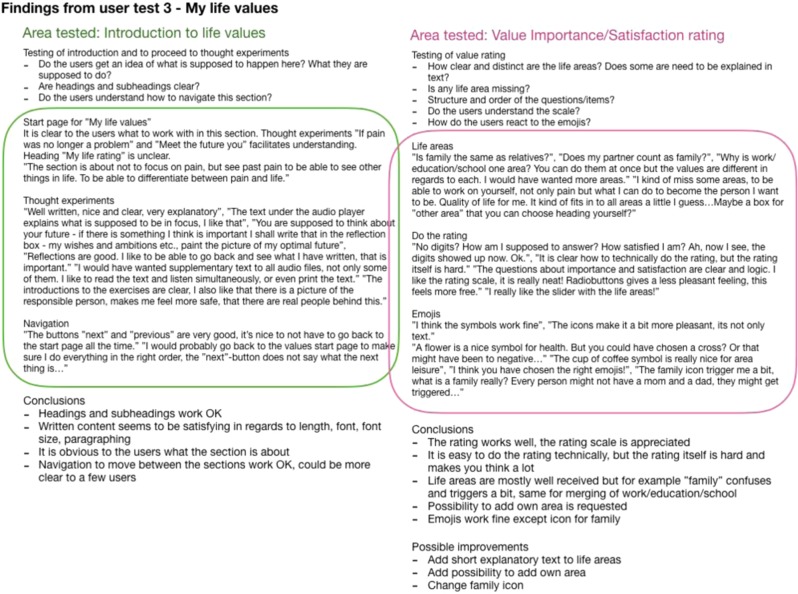
Example of documentation of results after alpha testing a feature of the solution (translated from Swedish to English).

Figure 3 shows an example of documented insights after testing a specific feature. The feature, content, or design was reiterated and re-tested until all alpha testers no longer found critical or blocking issues, and the development team considered all core features and functionality to be implemented. The solution was then considered a beta-ready product.

### Beta testing (phase 2)

The vast majority of patients included in the beta testing reported being satisfied with the treatment content and found it sufficiently short, comprehensible and with language not being too difficult. Many patients appreciated being able to listen to all written content. However, some individuals perceived the amount of exercises overwhelming and suggested a reduced number. Being able to save and revisit own written reflections was also suggested to be highly meaningful to monitor change and progress. Some beta patients appreciated the flexible format of the treatment program (being able to choose the order of work sections and exercises) while others preferred a clearer direction on when to do what.

The values-section was perceived as the least satisfying to many patients in the beta testing, due to difficulties understanding purpose and function, instructions and/or exercises.

Most patients found the treatment motivating, but what feature or part of the treatment they reported motivated them the most varied between the exercises, value work, and contact with a therapist.

**Fig. 4:**
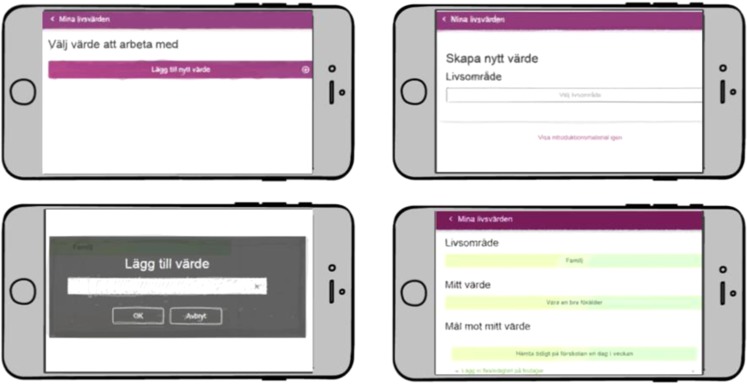
Each step for formulation of values at end of alpha testing, approved by all alpha testers (the native version).

**Fig. 5:**
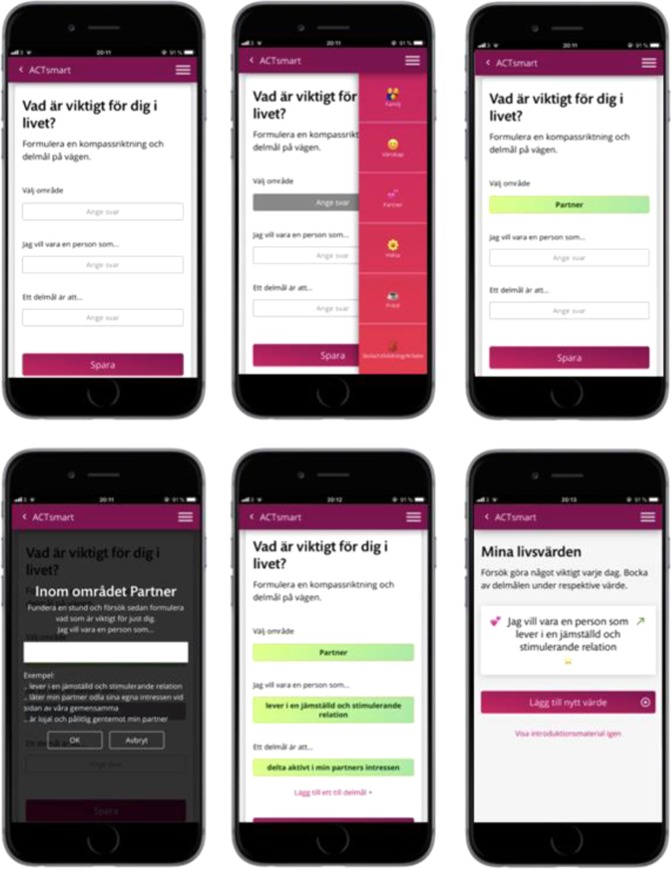
Each step for formulation of values after iterations based on beta user insights (the native version).

Insights for future development to move ACTsmart from beta-ready product to clinical trial ready product was gathered by the development team based on beta testing. Of these, some were considered research questions to address in future studies, while others were used to guide immediate improvements. Examples of the latter were the suggestion to have the possibility to see previous reflections on exercises, to clarify the expected level of work effort during each week of the treatment program, and how to restructure and simplify the values section. An example of an iteration post-beta trial, based on beta user insights can be seen in Figs 4 and 5, where Fig. 4 shows the steps through formulation of values after approval of alpha testers, while Fig. 5 shows the steps through formulation of values and the iterations that followed based on post beta interviews. The changes included restructuring and simplifying the texts as well as adding more steps in the formulation of life values, goals, and sub-goals.

Therapists involved in the beta testing described lacking a clear structure to guide the patients through the treatment. Also, they reported a number of therapist tasks being time consuming, such as searching for specific content referred to by the patient. Still, all therapists involved in the beta testing expressed that they found ACTsmart to be mostly user friendly and wanted to continue using it as a clinical tool. Implications for future development based on therapist beta-testers were for example to make treatment content searchable from therapist interface, develop the possibility to send both chat messages and reminders from the same platform and to improve clarity in the treatment manual. Detailed results of individual UX-interviews post treatment, as well as implications for further development and testing, are summarized in Table 1 (patients) and Table 2 (therapists).

**Table 1:** Key patient end-user insights and implications from UX-interviews post beta testing.

Acceptability question	Beta user insight	Implications
Satisfaction with treatment content	• Positive to the text-based content (sufficiently short, comprehensible, not too psychological). • Appreciated being able to listen to all written content. • Most participants found the exercises helpful. • The amount of exercises was overwhelming. • Annoying to not be able to see own previous reflections of exercises.	• Develop possibility to see previous reflection on exercise. Alpha-test and re-iterate. • Run beta trial with reduced amount of exercises. • Run beta trial that starts with fewer exercises, dispensing more during the course of the treatment.
Satisfaction with treatment format	• Some appreciated the free format and had no difficulties navigating through the intervention, others lacked structure and clarity. • One participant felt stressed by not knowing how much time or effort that was required. • Some participants found it hard to know when to proceed from theory and exercises to values and exposure.	• Develop “bulletin board” on the start page with “tip of the week” and current treatment week. Alpha-test and re-iterate. • In therapist treatment manual clarify expected work effort during the current week with instructions to inform the participants continuously. Alpha-test and re-iterate.
Satisfaction with values section	• Most respondents experienced the values section as difficult initially. • Some participants found layout and examples helpful while others found it to be even more confusing and unclear. • A few participants did not understand the connection between test of prioritized life values and later values work. • Some found formulation of values and the possibility to tick goals and steps as motivating while others did exposure/behavior change without ticking goals and steps in the application.	• Restructure and simplify values section. Alpha-test and re-iterate.
Treatment’s ability to motivate	• Many found the exercises motivating. • Many found the values work helpful as direction for change. • Some emphasized the possibility of receiving support from their therapist as motivating. • Most positive with ACT as form of treatment.	• No immediate development, alpha-test or iterations planned based on this feasibility area.

**Table 2:** Key therapist end-user insights with implications from UX-interviews post beta testing.

Acceptability question	Beta user insight	Implications
Satisfaction with treatment format	• Lacked a clear path that guided participants through treatment. • Too much work directed at suggesting to the patients what to do next.	• Develop “bulletin board” on the start page with “tip of the week” and which treatment week it is. Alpha-test and re-iterate.
Intent to continue use in clinical work	• All therapists wanted to continue using ACTsmart as a clinical tool, as single treatment contact and/or supplement to face-to-face treatment to give/monitor homework and/or reduce number of sessions face-to-face.	• No further development, alpha test or iterations planned based on this feasibility area. • Implementation studies in various clinical settings with varying levels of expertize in clinicians. • Studies on blended care approach combining face-to-face treatment with ACTsmart.
Use of therapist time	• Time consuming to scroll through treatment content to answer content-specific questions. • Inefficient to send text messages from different platform. • Inefficient to need to log in to see new patient activities in treatment, notification function suggested.	• Make content available and searchable from therapist interface. Alpha-test and re-iterate. • Investigate regulatory possibilities to send text messages from treatment platform. • Investigate regulatory possibilities to use push notifications (to patients). • Decision to not notify therapists on all treatment activity by push notifications due to protection of work/life balance.
User friendliness	• Therapists perceived design, format and most content user-friendly for participants but not the on-boarding process. • Therapists perceived the expected work effort for the patients unclear. • Therapists suggest emphasizing that the treatment progress requires patient engagement, e.g. repeated exercises.	• Develop process for on-boarding, including expected work load and level of engagement for patients. Alpha-test and re-iterate. Beta test in clinical trial.
Supports communication with patients	• Sparse communication from some (low activity) patients. • Lacked total overview of patient’s treatment activity due to immaturity of therapist interface which complicated providing specific/relevant feedback.	• Further technical development of therapist interface. Alpha-test and re-iterate. • Rewrite treatment manual with actions to identify and reach inactive patients at earlier stage. • Develop technical solution to flag uncompliant patients. • Alpha-test and re-iterate the above. • Beta-test in clinical trial.

Of the 31 patients that started treatment during beta testing, 28 (90.3%) completed treatment according to our pre-defined criteria of completion. Patients completed on average 84% of treatment content, and 26 (84%) of the patients formulated values and reported behavior changes towards at least one value. On average, patients sent 6.2 chat messages to their therapist (range 0–18). Many patients requested to have access to the application and material post treatment but only 6 (21%) of completers logged in during the 12 months they had access to the system after the end of the treatment period. Usage data is summarized in Table 3.

**Table 3:** Quantitative feasibility data from beta testing.

Feasibility area	Result
Usage, *n* = 31	
Completion, *n* (%)	28 (90%)
Completed treatment content^a^, *m* (median)	84% (90%)
Formulated values and reported valued action, *n* (%)	26 (84%)
Number of chat messages to therapist, *m* [range]	6.2 [0–18]
Logins after end of treatment among completers, % (*n*)	6 of 28 (21%)
Practicality, *n* = 31	Mean [range]
Therapist minutes per patient	127 [17–254]
Text messages reminders outside platform (per patient)	1.94 [0–6]
Therapist phone calls (per patient)	0.29 [0–1]
Technical feasibility	*n* (percent)
No of cases that required second line^b^ support	18
Regarding patient interface	11 (61%)
Regarding therapist interface	7 (39%)
Reason for support need	
Technical bug	12 (67%)
User error	4 (22%)
Missing function in therapist interface	2 (11%)
Device used, *n* = 16	*n* (percent)
Smartphone only	7 (44%)
Smartphone and computer	4 (25%)
Smartphone and tablet	2 (13%)

^a^Refers to completion of all available content, text-based or exercises.

^b^First line support was the supervising psychologist, second line support was technical staff.

All therapists interacted with patients and treatment content in a regular browser on a desktop computer (no additional software was required). Double authentication was used via the therapist’s smartphone.

In total, therapists spent on average ~2 h per patient throughout the treatment, and on average ~16 min per patient/week (range 2–32). Therapists sent in total 66 text message reminders outside the platform (push notifications were not possible at this stage of the development) (*m* = 1.94, range 0–6) and made in total ten phone calls (*m* = 0.29, range 0–1) during the course of the treatment. The most commonly used device by patients was a smartphone (44%), and the majority of the work performed in treatment was carried out at home (57%). In the beta testing, patients reported no technical issues that were specific for a certain device or brand. For further data on practicality, see Table 3.

## Discussion

ACTsmart is feasible with regards to usage, acceptability, and practicality, which warrants subsequent studies to evaluate the effect of this digital intervention. Importantly, the feasibility results suggest that the structure of the intervention was well received but also provided extensive feedback on what could be further improved to meet the needs of the end users before moving forward to test the effects in clinical trials. Positive aspects of the treatment and the digital solution that was reported by the end users was the micro-learning format, the use of everyday language, the opportunity to choose whether to read or listen to content, as well as acceptance of ACT when delivered digitally via smartphone. Aspects of the treatment that were reported as less satisfactory was for instance the inability to save and revisit own responses, as well as difficulties in planning how much work to put in and when to do what work. Also, the amount (or dose) of the material and exercises seemed to be important; too little was perceived as insufficient and too much was considered overwhelming. Moreover, although many of the patients in beta-testing requested continued access to the treatment material after the regular treatment phase was over, few continued to log in after the active treatment period. The therapist contact, as well as a clear treatment time frame, seems to be important to patients’ acceptability and attrition in treatment. However, this should be addressed in further studies by for example comparing guided and unguided treatment.

The intervention and delivery format were well received also among the therapists, and they reported several benefits when using ACTsmart in their clinical work. For example, the digital format promoted continuous work with behavior change more clearly compared to the usual face-to-face sessions. However, therapists involved in the present study wanted a more detailed therapist manual. Also, it was reported that further developments should make it easier to navigate within the treatment content to be able to more quickly respond to content-specific questions from patients.

In summary, many of the issues that came up in both alpha and beta testing were universal in nature, and did not specifically reflect chronic pain and associated symptoms, and should be considered when developing digital solutions aimed at behavior change or management of other chronic diseases.

The present study also illustrates the usefulness of the mHealth Agile Development & Evaluation Lifecycle, where the agile process allowed for a continuous development of the technical solution and the intervention until satisfying levels of acceptance and practicality was reached. The first draft of the treatment and technical solution that was outlined by the development team in the preparation phase changed radically during alpha testing, while beta-testing mainly provided information regarding minor adjustments and fine tuning, testing of practical and technical aspects that was not detectable in the alpha test phase, as well as generating ideas for future research questions. To go directly to clinical trial with a solution that has not been previously tested or guided by actual end users could result in a costly, time consuming and non-user-friendly solution that might risk poor retention rates. There might also be a heightened risk that a potentially effective treatment is falsely rejected when poor results are due to a poor technical solution, or an unsatisfactory delivery of treatment.

In line with previous findings, the present study suggests that smartphone treatment can reduce therapist time spent per patient.^26–28^ Furthermore, this treatment format can bring the behavior change program closer to the patients’ everyday lives as it prompts and supports both practice and use of target behaviors in real-life situations where the behavioral change takes place.^29^ Also, mHealth solutions can facilitate a better understanding of patient behaviors through continuous data collection with high ecological validity.

A few limitations to the present study should be considered. The alpha testing was based on a convenience sample with highly motivated patients and therapists, and the beta testing utilized a sample of self-referred patients with an interest in undergoing a digital self-management behavioral intervention. However, the self-referred beta sample has a similar pain duration as participants in previous research recruited from a tertiary pain clinic.^30,31^ Still, it is yet unclear to what extent the feasibility results are generalizable to the broader pain population.

A preliminary efficacy testing of the treatment outcome is required to evaluate the effects of an intervention. Also, larger clinical trials with different samples are needed to scientifically assess the utility and external validity of ACTsmart, as well as the change mechanisms (mediators and moderators of treatment outcome) including for whom this intervention may be useful. In addition, the required level of therapist competence and the need for therapist support to obtain satisfying treatment effects, should be addressed in future research. Furthermore, cost-effectiveness and dose-response relationship are important research objectives.

Although large clinical trials (RCT:s) have traditionally been the method of choice for efficacy testing, research methods compatible with agile development should be considered. Studies addressing the utility of specific treatment components, change mechanisms, and tailored interventions may benefit from utilizing the mHealth Agile Development & Evaluation Lifecycle in combination with evaluation methods such as single case experimental design, A/B testing, small group iterations, and continuous UX testing. Further development and evaluation of the utility of the specific components and technical functions within ACTsmart requires a series of optimization studies, which may benefit from applying approaches that allows close monitoring of individual trajectories and relationships between interventions and change processes. Such bottom-up approach will facilitate the development of tailored or flexible treatment programs, where specific components are combined to address individual needs. Individual-level data may shed light on important aspects such as dose-response relations and mechanisms of change (moderated mediation), which are critical to empirically driven personalized treatment and improved treatment effects on a larger proportion of patients.

Based on feedback from therapists involved in the beta testing, future research should also explore the possibility to integrate ACTsmart with face-to-face treatment. Combining standard treatment with a digital intervention may have several benefits. For health care organizations, digital interventions may facilitate standardized care across therapists or health care units; quality assurance through improved protocol adherence and a minimal deviation from the empirically supported practice (therapist drift). Furthermore, digital solutions may enhance treatment compliance and support behavior change between sessions.

To conclude, the results and completion of the first three phases of the mHealth Agile Development & Evaluation Lifecycle has provided the opportunity to further optimize ACTsmart as well as validate that the form of delivery is feasible and acceptable. These steps are crucial before moving ACTsmart to clinical trials that evaluate the effects and change mechanisms of the therapeutic intervention.

## Methods

### Procedure and design

**Fig. 6:**
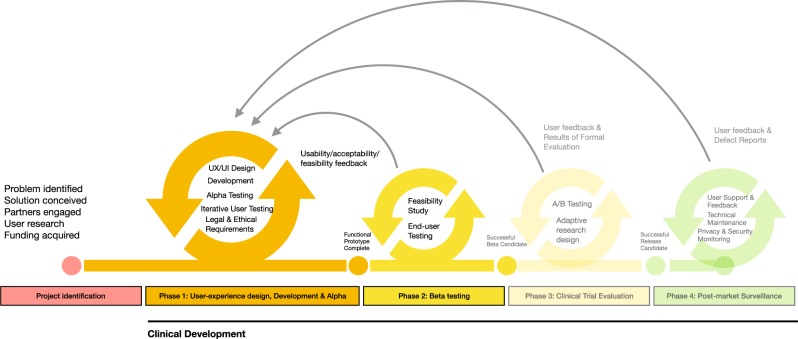
The mHealth Agile Development & Evaluation Lifecycle (Wilson et al.^25^) adapted with permission to the ACTsmart development project.

Within the present study the three first phases (0–2) of the mHealth Agile Development & Evaluation Lifecycle^25^ were completed. In the present study phase zero will be described briefly, while phase one and two are described in more detail. Figure 6 illustrates the lifecycle, adapted to the ACTsmart development project. In phase zero, the project identification phase, the agile and user centered development method Lean UX^32^ and scrum methodology^33^ were used as project management approaches. Phase one and two were divided into five sprints. Each sprint had specific objectives and continued during ~30 days. The project leader compiled all proposed changes for the solution and prioritized among possible functionality enhancements. Early in phase one, the decision was made to build an independent, cloud-based and flexible technical solution, rather than further develop an existing platform, to maximize flexibility in the development. Also, it was decided to focus on the patient interface during phase one, and to prepare the patient interface for beta testing in phase two. Consequently, the therapist interface was still rudimentary when the beta testing/clinical feasibility trial was conducted.

### Alpha testing

Alpha testing is an acceptance testing preferably carried out with potential end-users. It involves simulating a real user environment by carrying out tasks that actual users might perform. The alpha test is made to identify as many potential problems as possible before releasing a product for beta testing, and can be performed on early versions of the product such as paper sketches, versions that lacks all features and on versions that is yet too unstable for reliable use. The alpha testing also gives preliminary end-user feedback, to get feedback when adjustments are still easy to make.

In phase one, nine individuals (age 19–65 years, 78% women) with complex chronic pain (potential end-users) were recruited for alpha testing from a tertiary care pain clinic after completing a standard face-to-face ACT-treatment. Alpha testing began with end-user interviews to inform personas and work flow ideas. Based on these nine interviews three personas were generated. The interface was then built based on the personas and continuously and repeatedly tested with the alpha users. See Table 4 for a detailed description of the test flow.

**Table 4:** Development and evaluation process of ACTsmart.

**Phase 0**	**Innovative**	**Organizational**				
	Identification of challenge. Envision of product. User research. Identify end-users. Identify target market.	Gather expertize and resources needed. Create project organization. Identify strategic/operative goals. Secure funding and resource allocation for phase 1. Define work model.				
**Phase 1**	**Organizational**	**Technical development**	**Content development**	**End-user insights**	**Alpha testing and re-iterations**	**Beta testing**
Sprint 0	Establishment of effect map with goals, target groups and user needs.	Identify basic needs and functionality. Set limitations for phase 1 (focus on patient interface). Make basic technical choices (independent, cloud based, flexible).		Interviews with clinicians specialized in chronic pain.		
Sprint 1	Definition of operationalized short-and long-term goals. Identification of external risk factors and strategies to prevent/manage the risks.	Decision to use Microsoft Azure cloud-based platform for both content and data collection. Multidisciplinary design studio session to generate ideas for functionalities.	Identification of types of content (text, audio, video).	Interviews with patients. Creation of protopersonas.		
Sprint 2		Production of HTML-prototype for weekly patient reported outcome measures.	Paper sketches for content structure. Production of first versions of written content.		HTML-prototype. Content structure sketches.	
Sprint 3		Creation of draft for system navigation and system levels.	Prototype for audio content. Prototype for written content. Draft of first animated video.		Weekly patient-reported outcome measures. Solution content. Solution structure.	
Sprint 4			Production of first draft of the structure and content of value module. Creations of more animations. Recording of audio tracks. First drafts of treatment illustrations.		First draft of value module. Animations. Audio tracks. Illustration drafts.	
Transition period		Creation of messaging function. Bug testing.		Collection of system generated data. Interviews with alpha testers. Interviews with clinicians.	First compound prototype of the application. Weekly patient-reported measures.	Planning of beta testing in clinical feasibility trial.
**Phase 2**	**Organizational**	**Technical development**	**End-user insights**	**Alpha testing and re-iterations**	**Beta testing**	
Sprint 0	Joint application for further funding. Resource allocation for phase 2.	First sketches of therapist interface. Creation of double authentication login.			Recruitment of end-users (adult pain patients) for beta testing.	
Sprint 1		Development of web prototype for therapist interface. Ongoing debugging. First- and second line technical support.		Draft sketches of therapist interface. Web prototype of therapist interface.	Start of 8-week beta testing (clinical feasibility trial).	
Sprint 2		Ongoing debugging. First- and second line technical support.	Preparation of UX-survey and UX-interviews with beta testers.	Web prototype of therapist interface.	Ongoing beta testing (clinical feasibility trial).	
Sprint 3		Production of differentiated access for therapist interface.		Web prototype of therapist interface.	In-depth beta UX interviews. Beta UX survey.	
Sprint 4		Workshop on handling of support and bugs. Completion of first version therapist interface.	Compilation of beta UX insights.	Patient interface.		
Transition period				Patient interface. Therapist interface.		

**Fig. 7:**
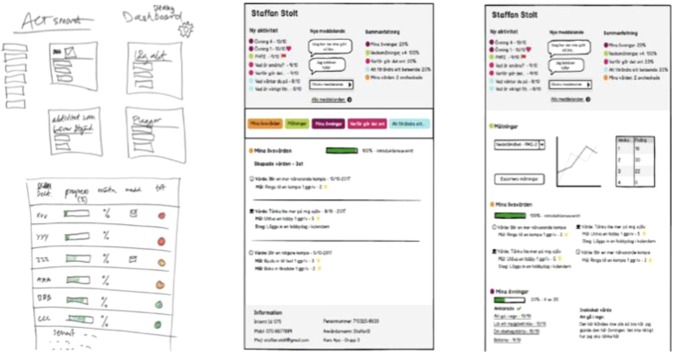
Paper sketches showing the evolution of the list of patients in active treatment in the therapist interface during alpha testing.

Six therapists were continuously involved in the alpha testing of the therapist interface which was carried out in the same way as with the patient alpha testers. See Table 4 for a detailed description of the test flow. Figure 7 shows an example of a development of one of the features in the therapist interface throughout the different phases of alpha testing.

### Beta testing

The alpha testing phase is followed by beta testing (phase 2), in which the intervention is tested by real users in a real environment. In the present study, the beta testing sample consisted of 31 individuals (87% women, aged 25–57) with chronic pain (mean pain duration 19.74 years, range 0.5–40 years) with no prior exposure to behavioral treatment for their chronic pain condition, actively requesting participation in a research study on internet-delivered ACT. In the beta sample, seven out of 31 (23%) underwent a UX-interview and 16 out of 31 (52%) answered a UX-survey. Four therapists trained in ACT and behavioral treatment of chronic pain delivered treatment during the beta testing.

After both alpha (phase 1) and beta (phase 2) testing were completed, preparation of the next phase included minor adjustments identified during the sprints and debugging. See Table 4 for detailed information on the development and evaluation of phase 0–2.

### Intervention

ACTsmart is based on the clinical treatment program developed at Karolinska Institutet and Karolinska University Hospital during the past 18 years. To date, the ACT based treatment program for chronic pain patients has been evaluated in nine clinical trials, including five RCT’s^31,34–37^ with results illustrating the efficacy of the protocol. Improvements are primarily seen in functioning/disability and psychological flexibility, with mostly large effect sizes. Results are consistent across all studies, supporting the external validity of the findings.

The overarching goal of the ACTsmart treatment is to improve functioning and quality of life by increasing the participants psychological flexibility, defined as the ability to act in accordance with life values in the presence of pain and distress.^38^ Psychological flexibility is established through promoting greater acceptance of negative inner experiences^22,31^ as well as increasing ability to observe, rather than being entangled with, thoughts and engage in valued action.^38,39^

**Fig. 8:**
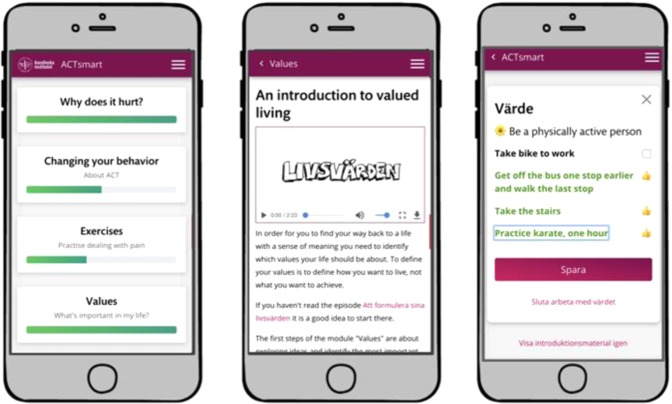
Screenshot of patient interface, home screen, introduction to values, and values work section.

**Fig. 9:**
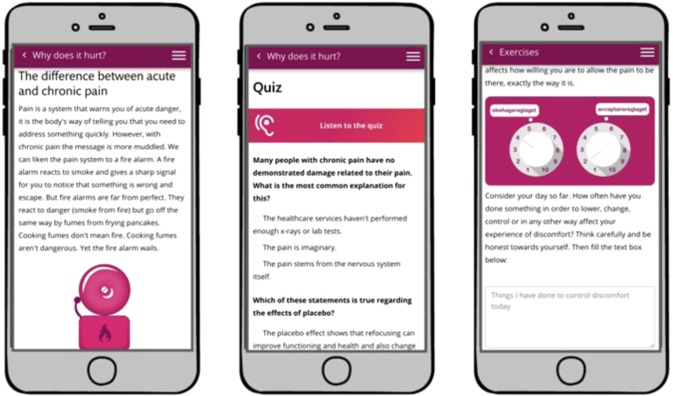
Screenshot of patient interface, educational content, and exercise.

In treatment, participants were encouraged through content, solution design and by their therapist to redirect behaviors and shift focus from avoiding or reducing pain and distress to act in alignment with values in the presence of interfering pain and distress. Thus, patients were encouraged to engage in value-based exposure. Treatment content was divided into different themes that roughly corresponds to the core processes of ACT;^38^ acceptance, creating distance to thoughts, creating distance to emotional and bodily experiences, noticing and changing behaviors, self-observation, and values. Content was also categorized depending on type; educational, exercise or value-work. See Figs. 8 and 9 for screenshots of the patient interface.

### Completion

The pre-defined criterion of treatment completion was the combination of (a) completing at least eight exercises, (b) having defined at least one formulated value, and (c) reporting behavioral actions towards that value. This criterion was chosen as exercises and values-work are based on experiential learning and therefore expected to be the active ingredients in treatment,^40^ in contrast to educational content that have the purpose of preparing for experiential learning.

### Intervention structure

The intervention was arranged in a micro-learning format, which is the combination of micro-content delivery and micro interactions that seeks to enable the user to gain knowledge and skills without risking information overload.^41^ Micro-content learning through a mobile device can give the user personal control and ownership of the learning process.^42,43^ All content in the treatment could be either read or listened to, in order to accommodate different preferences and needs.

### Data collection

The present study was part of an open-label pilot trial with one intake. The pilot trial was approved by the Regional Ethics Committee in Stockholm, Sweden 3 November 2015 (2015/1638-31/2) and followed the Helsinki declaration. The trial was registered at clinicaltrials.gov at 17 November 2017 with registration number NCT03344926. Participants provided written informed consent prior to enrollment in the study.

### Data security

Treatment was delivered on a secure platform and log in required double authentication from both patients and therapists. Data storage differed depending on the type of data collected. Personal data that could be traced back to a specific individual was stored on a secure server and anonymized data was stored in a cloud solution using a cloud storage provider that was certified according to security and auditing standards ISO 27001 and SAS 70/SSAE 16 as well as connected to the Privacy Shield principles of data processing and thus complied with the European legislation (GDPR) requirements for the processing of personal data. Security was also managed through various levels of access, for example therapists only had access to data regarding their specific patients while research admin had access to all patient data.

### Alpha testing

Data collected during the alpha testing consisted of qualitative data on user behaviors and experiences. All tests were documented by the test leader and then brought back to the development team to guide further development.

### Beta testing

In the beta testing, system generated quantitative user data was extracted during the course of the treatment/testing. Qualitative UX-data was derived from interviews with seven patients (23%) and all four therapists post treatment/testing as well as through a UX-survey that was completed by 16 (52%) of the patient beta testers. Data was compiled, organized into themes, and sorted for immediate re-iteration, future research questions to address or future development.

The main purpose of the beta testing was to examine feasibility. The variables of interest were acceptability, usage and practicality. Acceptability concerns to what extent the intervention program and format of delivery is suitable, satisfying, and attractive to the users, both recipients (patients) and deliverers (therapists).^44^ Usage data provides information on to what extent the participants accept the treatment, and at what level participants use the solution throughout the course of the treatment. Practicality refers to the possibilities of carrying out the intervention based on existing means, resources, and circumstances and without outside intervention^44^ as well as technical feasibility.

Acceptability data was collected pre-treatment (during alpha testing) as well as post treatment (as part of the beta testing) in qualitative UX-interviews. The alpha testing investigated user friendliness, comprehensiveness, and usability and led to iterations and continuous retests until they met a satisfactory level for all alpha testers before the intervention was ready for beta testing. Beta testing investigated satisfaction with treatment content, satisfaction with treatment format, satisfaction with values section as well as the treatment’s ability to motivate the user. Qualitative acceptability data was also collected from therapists in a post-treatment focus interview on satisfaction with treatment format, intent to continue use, use of therapist time, user friendliness as well as if the intervention supports patient communication.

Usage data was generated by the solution through completion rate, number of chat messages to therapist as well as post treatment logins.

Practicality data was collected during treatment and included therapist minutes per patient, text message reminders and phone calls from therapist to patient. Technical feasibility, as part of the practicality aspect, concerned how well the system worked in real-life situations through the need for technical support and reason for the need of technical support. Post-treatment quantitative data was collected in a UX-survey investigating device used, and in what working context patients engaged in treatment.

### Reporting summary

Further information on research design is available in the Nature Research Reporting Summary linked to this article.

## Supplementary information

Reporting Summary

## Data Availability

The dataset generated during the current study is not publicly available due to restrictions in the ethical permit, but may be available from the corresponding author on reasonable request.

## Code availability

Given the descriptive nature of the analyses no custom code or mathematical algorithm was used in this study.
